# Association by Spatial Interpolation between Ozone Levels and Lung Function of Residents at an Industrial Complex in South Korea

**DOI:** 10.3390/ijerph13070728

**Published:** 2016-07-19

**Authors:** Soon-Won Jung, Kyoungho Lee, Yong-Sung Cho, Ji-Hee Choi, Wonho Yang, Tack-Shin Kang, Choonghee Park, Geun-Bae Kim, Seung-Do Yu, Bu-Soon Son

**Affiliations:** 1 Environmental Health Research Division, National Institute of Environment Research, 42, Hwangyeong-ro, Incheon 22689, Korea; melon777@korea.kr (S.-W.J.); lm8080@korea.kr (T.-S.K.); whoispch@korea.kr (C.P.); mykgb@korea.kr (G.-B.K.); sdyu@korea.kr (S.-D.Y.); 2 Occupational Epidemiology, Samsung Health Research Institute, Samsung Electronics, Giheung City 17113, Korea; 3 Research Development and Education Division, National Institute of Chemical Safety, 90, Gajeonbuk-ro, Daejeon 34111, Korea; yscho72@korea.kr; 4 Department of Environmental Health Science, Soonchunhyang University, 22, Soonchunhyang-ro, Asan-si 336-745, Korea; jhchoi@sch.ac.kr; 5 Department of Occupational Health, Catholic University of Daegu, 13-13, Hayang-ro, Daegu 38430, Korea; whyang@korea.kr

**Keywords:** spatial interpolation, ozone, lung function, kriging

## Abstract

Spatial interpolation is employed to improve exposure estimates and to assess adverse health effects associated with environmental risk factors. Since various studies have reported that high ozone (O_3_) concentrations can give rise to adverse effects on respiratory symptoms and lung function, we investigated the association between O_3_ levels and lung function using a variety of spatial interpolation techniques and evaluated how different methods for estimating exposure may influence health results for a cohort from an industrial complex (Gwangyang Bay) in South Korea in 2009. To estimate daily concentrations of O_3_ in each subject, four different methods were used, which include simple averaging, nearest neighbor, inverse distance weighting, and kriging. Also, to compare the association between O_3_ levels and lung function by age-groups, we explored ozone’s impacts on three age-related groups: children (9–14 years), adults (15–64 years), and the elderly (≥65 years). The overall change of effect size on lung function in each age group tended to show similar patterns for lag and methods for estimating exposure. A significant negative association was only observed between O_3_ levels and FVC and FEV_1_ for most of the lag and methods in children. The largest effect of O_3_ levels was found at the average for the lung function test day and last 2 days (0–2 days). In conclusions, the spatial interpolation methods may benefit in providing individual-level exposure with appropriate temporal resolution from ambient monitors. However, time-activity patterns of residents, monitoring site locations, methodological choices, and other factors should be considered to minimize exposure misclassification.

## 1. Introduction

Air pollution exposure assessment is a crucial component to investigate the relationship between air pollution and health effects in epidemiological studies. As the magnitude of the health effects of ambient air pollution often relies on exposure assessment methods, the method for estimating exposure is an important factor to determine the actual or potential exposure level of humans. The most desirable method for estimating personal exposure is to measure directly through personal monitoring [1]. However, since direct measurement of exposure at the individual level can be time consuming and expensive, in large-scale epidemiological studies it can be commonly estimated by fixed ambient air pollution monitoring data. Also, most epidemiology studies use data spatially aggregated at the area level because of data limitations.

The advantage of monitoring data includes the ability to use existing data and to cover a large spatial area [2]. Previous epidemiological studies for air pollution have assigned exposures using data from a few nearest air monitors and used the exposure as a surrogate for the actual personal exposure [3,4]. However, this approach may cause uncertainty such as exposure misclassification, and may underestimate or overestimate the health effects of air pollution because it does not reflect the spatial heterogeneity of individuals [5,6].

Recently, spatial interpolation is increasingly being used to assess adverse health effects associated with environmental risk factors. Advances in geostatistical methods and geospatial technologies based on geographic information systems (GIS) have made it possible to improve air pollution exposure assessments. Specifically, a number of studies have been conducted to estimate the relationship between air pollution and health outcomes using spatial methods such as proximity models [7,8], geostatistical methods [9,10] and land-use regression models [11,12]. In particular, to minimize exposure misclassification, advanced interpolation methods have been applied to better estimate a population’s or individual’s exposures to air pollution. Kriging is one of these interpolation methods, which is developed based on statistical techniques in geostatistics for optimal spatial prediction at unobserved locations [13,14].

The short-term exposure effects of ozone have been demonstrated to cause lung function impairment, lung inflammation and respiratory symptoms [15]. In addition, high O_3_ concentrations have been associated with adverse effects on respiration and lung function [16]. While many studies have focused on the characteristics of surface ozone concentration and distribution, relatively few have investigated how this pollutant affected health outcomes. Also, almost no investigations have utilized improved methods for estimating exposure for the association between ozone and health outcomes.

In this study, we investigated the association between ozone level at an industrial complex in South Korea and lung function with two objectives, one of which is estimating and comparing ozone exposures using four different methods including simple averaging across all monitors in the study area, spatial interpolation by the nearest monitoring station, inverse distance weighting and ordinary kriging and the other of which is evaluating how different methods for estimating exposure influence health outcomes.

## 2. Materials and Methods

### 2.1. Study Area and Subjects

The study area is a major national industrial complex (Gwangyang Bay) where petrochemical and steel-related industries such as steel mills and a thermal power plant are densely placed. This area is a geographically closed coastal area where air pollutants become stagnant and do not spread into the atmosphere. It is well established from the national environmental project [17] that surface ozone is a secondary air pollutant, which affects human health.

**Figure 1:**
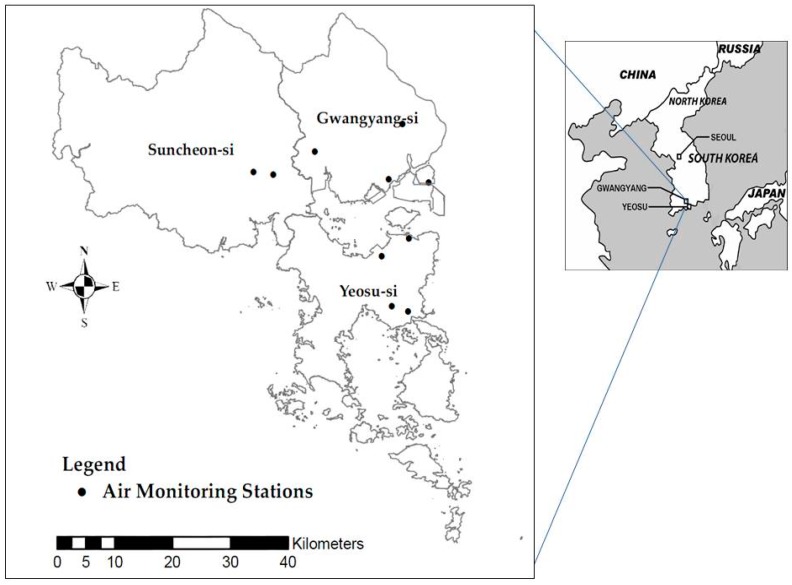
The locations of the study area and air monitoring stations.

Information concerning demographic characteristics, and residential and medical histories was investigated. A total of 2283 participants who had registered accurate residential addresses and undergone a pulmonary function test were recruited in 2009. Figure 1 shows the study area and location of the fixed monitoring stations. This study was approved by the Institutional Review Board of Soonchunhyang University (IRB ID: 2007-15-02).

### 2.2. Ambient Ozone Exposure Estimation

Concentrations of O_3_ were measured for every hour of every day by an ultraviolet photometric analyzer using 10 ambient air monitoring stations located in Gwangyang Bay. We obtained hourly O_3_ data to analyze their association with the lung function of the study subjects. For statistical analysis, the maximum daily 8 h moving average of the ozone data was calculated. 

To estimate daily concentrations of O_3_ in each subject, four different methods were used. Individual O_3_ exposure was assigned the values of the average of all monitors in the study area (Method 1), the predicted concentration observed at the nearest monitoring station (Method 2), inverse distance weighting (IDW) proportional to the distance from the estimated value (Method 3), and kriging were used to quantify the tendency of the differences between values at points to increase with distance (Method 4). Method 1 assigns exposure as the average value of all monitors in the study area. We calculated the average of the values from 10 monitors and assigned that value to the subjects as the same level of ozone exposure. Method 2 selects the value of the nearest monitor and does not consider the values of neighboring monitors at all. In this study, each subject was assigned the ozone concentration level of the single monitor nearest to the subject’s residence. Method 3 is the simplest interpolation method. The IDW interpolation method is based on the assumption that unknown values are influenced more by nearby points than far away points. The interpolation weights of each monitor’s values are calculated as a function of distance between the observed values of a sample site and the site at which the prediction is sought [18].

The observed values that are closer to the sample point of interest are more heavily weighted; however, a large search window can still preserve some of the local variations in pollutant levels [19]. To predict a value for location of subject’s residence, the data from all monitors within the Gwangyang Bay area were included in the IDW interpolation for O_3_. We utilized λi = 1/di as a weighting factor for monitor i, where di is the distance between monitor i and the point to be predicted [19]. Kriging is an interpolation technique that estimates values at unmonitored locations from observed values and semi-variograms. IDW uses a simple algorithm based on distance, but the kriging method (Method 4) was weighted based on spatial autocorrelation, which was determined from the variogram developed by the spatial structure of the data. Kriging requires the estimation of the empirical semi-variogram, based on a theoretical semi-variogram function such as spherical, gaussian and exponential models [20]. We used ordinary kriging with a spherical model to estimate the daily O_3_ concentration [21]. The spherical variogram is one of the most commonly chosen forms, and the ordinary kriging algorithm takes advantage of the assumptions made about an unknown constant mean value and is appropriate for our data.

### 2.3. Cross-Validation

In order to ensure the validity of the values predicted by the interpolation methods we performed cross-validation. Each air monitor’s ozone value was excluded, and then the remaining monitor values were used to estimate the ozone value at the excluded monitor location. To evaluate the quality of prediction by interpolation methods and similarity between the measured and predicted values, the root mean square error (RMSE) was calculated [22]. The consistency of the measured and predicted values was applied with the coefficient of divergence (COD). A COD of zero implies there are no differences between measured and predicted values, while COD values approaching one indicates maximum differences [23]. The RMSE and COD are defined as:

(1) $$\text{RMSE}=\sqrt{\frac{1}{p}\overset{p}{\underset{i=1}{\sum }}{\left(x_{ij}-x_{ik}\right)}^{2}}$$

(2) $$COD=\sqrt{\frac{1}{p}\overset{p}{\underset{i=1}{\sum }}{\left[\left(x_{ij}-x_{ik}\right)/\left(x_{ij}+x_{ik}\right)\right]}^{2}}$$

where X_ij_ and X_ik_ represent the average concentrations of the measured and predicted ozone for day i, and p is the number of observations.

### 2.4. Lung Function Tests

The lung function was measured with the forced vital capacity (FVC) and forced expiratory flow in 1 s (FEV_1_) methods during the morning hours in indoor buildings. These measurements were performed by experienced technicians in accordance with the American Thoracic Society criteria [24]. Each subject was asked repeated measurements no more than five times until the best result appeared. Pulmonary function was performed using a spirometer (Spirovit model SP1, Schiller, Ottobrunn, Germany).

### 2.5. Statistical Analysis

We estimated O_3_ concentrations at the study subjects’ locations for the lung function test day (0 day), 1 day before the lung function test (1 day), 2 days previous (2 days), average of the test day and previous day (0–1 day), average of the last 2 days (1–2 days), and average of the test day and last 2 days (0–2 days). In order to compare the associations between O_3_ levels and lung functions by age-group, the ozone’s impacts on three age-related groups were explored with children (9–14 years), adults (15–64 years), and the elderly (≥65 years). In this study, multivariable regression analysis was used to determine FVC and FEV_1_ against predicted values of ozone at each lag and to assess the association of ozone levels with pulmonary function (FVC and FEV_1_) by the four different methods for estimating exposure. A *p*-value < 0.05 was considered significant for all analyses. The data were analyzed using the R3.0.1 (R Foundation for Statistical Computing, Seoul, Korea) and SAS 9.2 (SAS Institute, Cary, NC, USA).

## 3. Results

A total of 2283 ozone exposures were estimated at the individual level from information based on the residential locations of the subjects. The summary descriptive statistics of O_3_ concentrations for each method are shown in Table 1. 

These summary statistics are the average across the 2283 subjects. The mean O_3_ values were 42.2 ppb from Method 1, 40.9 from Method 2, 41.4 from Method 3 and 41.7 from Method 4. The average values of estimated O_3_ exposures were not significantly different in each method. The largest variation in spatial concentration among the four methods was nearest the monitors (Method 2). Table 2 shows the mean and range levels of daily max 8 h moving average of O_3_ concentrations on each day, and the lung function test was performed for 1 lag, 2 lag, 0–1 lag, 1–2 lag and 0–2 lag by the four different methods for estimating exposure. 

Ranges of concentrations of ozone were 28.3–85.9 ppb (Method 1), 24.0–96.4 (Method 2), 24.0–93.9 (Method 3), 23.9–98.2 (Method 4). The concentrations of ozone on the day of the lung function test (0d) were the highest in all methods except for Method 2 (nearest monitor). We found that the estimated ozone exposure from kriging showed more excellent cross-validation results than those from the other interpolation methods from the cross-validation analysis. The RMSE indicated the difference between observed and predicted ozone concentration for kriging was the lowest. The COD value for kriging was less than those of nearest monitor and IDW (Table 3). 

A summary statistic of the demographic characteristics and lung function measurements of the study populations is presented in Table 4. The mean age of subjects was 41.8 year, and 56.9% were female. The mean FVC and FEV_1_ of each age group were 2.26, 1.92 in children, 3.23, 2.69 in adults and 2.39, 1.82 in the elderly, respectively (Table 4).

**Figure 2:**
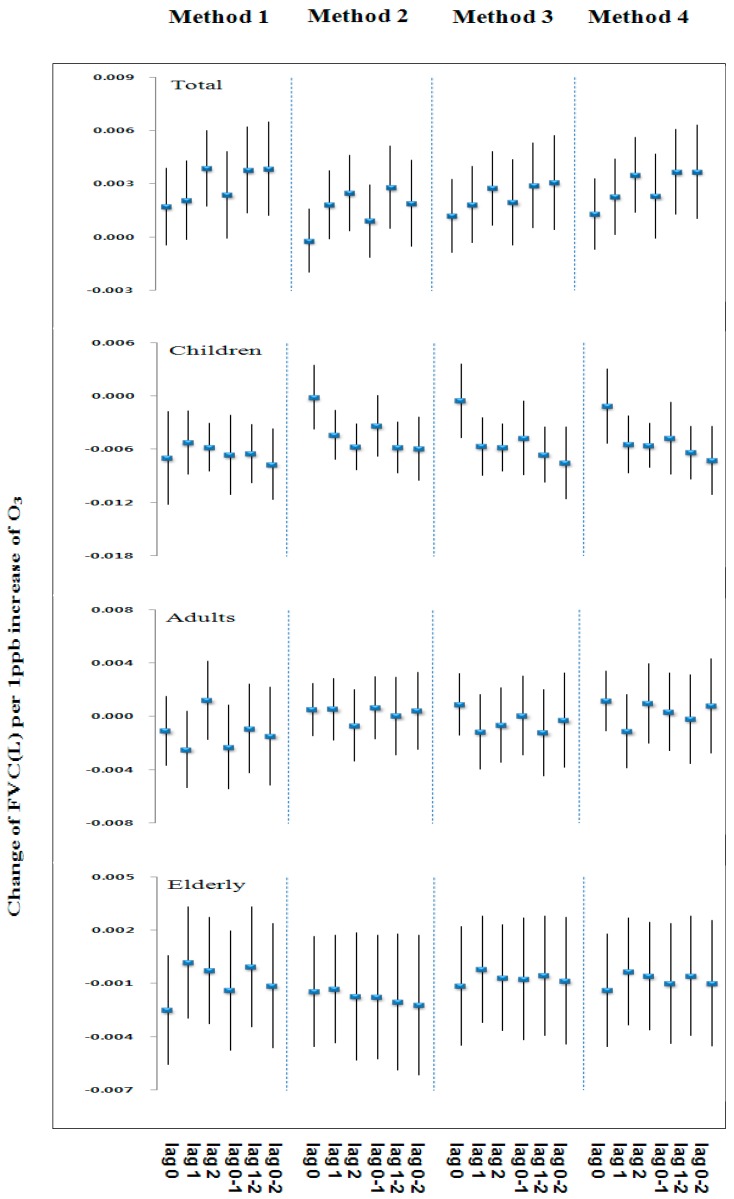
Change of FVC (L) by O_3_ concentration for various lags and methods for estimating exposure.

**Figure 3:**
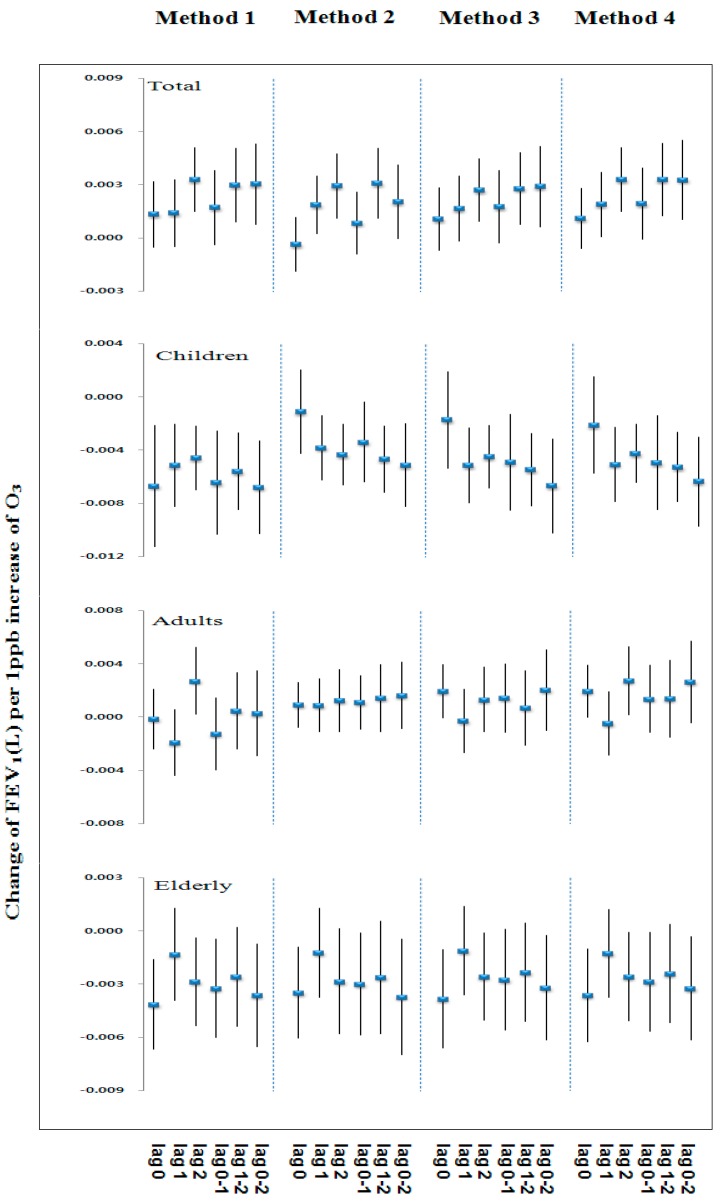
Change of FEV_1_ (L) by O_3_ concentration for various lags and methods for estimating exposure.

Figure 2 and Figure 3 show similar patterns of changes in effect size on FVC and FEV_1_ associated with exposure to 1 ppb increase of ozone concentrations by lag and the four methods for estimating exposure in each age group. The overall change of effect size on lung function in each age group tended to show similar patterns for lag and the methods for estimating exposure. Table 5 and Table 6 show the estimated changes in FVC and FEV_1_ for an increase to the inter-quartile range of O_3_ concentrations by the four methods and age groups. In children (age 9–14), the significant negative association were observed between O_3_ and FVC and FEV_1_ for most of the lag and the methods. 

The largest effect of O_3_ was found at the average of the lung function for the test day and last 2 days (0–2 days). In adult (age 15–64), the effect of O_3_ on reduction in FVC and FEV_1_ were not significant for any lag and the methods. In the elderly (age ≥ 65), the negative association between FEV_1_ and O_3_ was significant except on 0 day, 0–1 day, but FVCs were not significant for any lag and the methods. 

## 4. Discussion

This study assessed the association between ozone exposure and lung function using four methods for estimating exposure. To minimize exposure misclassification, we tried to estimate more accurate exposure levels by using the spatial analysis technique in the exposure assessment of epidemiological studies. Although other researchers have employed a similar method to estimate individual levels of exposure to air pollution, they did not use such approaches to compare the lung function effects of ozone in different age groups [25,26,27]. In addition, no investigations have been done utilizing interpolation methods for estimating exposure to identify the association between ozone and health outcomes in Gwangyang Bay.

In this study, we developed better exposure estimates through the application of a kriging method among the four interpolation methods. Previous studies with the application of interpolation methods have suggested the similar associations. A study by Son et al. predicted individual levels of exposure to air pollution using the four different approaches (average across all monitors, nearest monitor, IDW and kriging) to identify the association between air pollution and lung function [28]. They found that the kriging method provided the most accurate estimates of exposures, and the magnitudes of health effect estimates were generally higher when using exposures based on averaging across all monitors or kriging. A simulation study by Kim et al. examined exposure prediction approaches such as the nearest monitor and kriging methods for PM_2.5_ affecting relative risk estimates for cardiovascular events in Los Angeles [29]. Their findings indicated that a kriging method provided more accurate exposure estimates because it had smaller average mean square prediction error. Liao et al. reported that daily kriging estimations of residential−level ambient PM concentrations were feasible on a national scale [21]. Jerrett et al. suggested that the health effects associated with exposure to PM_2.5_ were approximately three times greater using the kriging approach than using the average ambient concentration approach previously employed in the American Cancer Society (ACS) cohort. This can be explained by the fact that the exposure estimation method used in estimating air pollution exposure may affect the results of epidemiologic studies [25]. However, there have been debates on which methods are most suitable for estimating air pollution from ambient monitors.

The advantage of regulatory monitoring network data includes the ability to use existing data and to estimate individual level exposure. This monitoring data would allow studies on the relationship between air pollution and health outcomes for times, locations, and pollutants for which monitoring data are limited or unavailable [2]. The Ministry of Environment (MOE) in Korea has generated hourly ambient air concentration data from 257 urban air quality monitors, including O_3_, PM_10_, NO_2_, SO_2_ and CO [30]. This monitoring network data provide good temporal resolutions, with hourly monitor coverage of 0.003 monitor/km^2^ at a national scale. Air monitoring stations have typically been installed in urban areas where population density is high [2,31]. For instance, the most densely populated Seoul, the capital of Korea, had hourly monitor coverage of 0.04 monitor/km^2^. Although fewer monitors were available in our study area, monitor coverage had a high spatial resolution of 0.01 monitor/km^2^.

Declines in FVC and FEV_1_ were significantly associated with ozone concentration in children. In this study, we assessed the relationship between changes in lung function and ozone exposure as estimated by the interpolation method. The results of this study show that there were no significant associations between ozone exposure and lung function in the whole subject, but a decrease in lung function was observed in children for most of the lag and methods. More specifically, the declines in FVC and FEV_1_ were significantly associated with most of the lag and methods, and the largest effect was observed for 0–2 days. Previous researches of decrease in lung function on O_3_ exposure in children have demonstrated similar results [32,33,34,35]. According to Chang et al., 1 ppb increase in O_3_ was significantly associated with decreased FVC 2.29 mL among 2919 students who lived in five school districts in Taipei [36]. Significant lag effects were observed in FVC for 0 day, 1 day and 2 days before the spirometry test. Son et al. reported that a 11 ppb increase (IQR) in O_3_ was associated with a decrease of 6.1% (95% confidence interval, 5.0% to 7.3%) in FVC and 0.5% (95% confidence interval, 0.03% to 0.96%) in FEV_1_ in lag 0–2 day, based on kriging exposures [28]. Moreover, ozone exposure is well documented in many studies to cause adverse lung injury effects, including reduced lung capacity, chronic obstructive pulmonary disease, and severe asthma exacerbation [37,38]. In this study, ozone level was calculated as the maximum daily 8-h moving average. The maximum daily 8-h moving average was computed by selecting the highest value among 17 daily 8-h moving average. Since lung function tests were performed in the morning, estimated ozone level for the lung function test day (0 day) may have implications for interpreting the association between ozone level and lung function.

It is well established that ground-level ozone is generated from the photochemical oxidation of NO_x_ emissions and volatile organic compounds (VOCs). The major sources of NO_x_ and VOCs are emitted during fuel combustion from automobiles, fossil fuel power plants and industrial processing. Our study area was a major industrial hub in Korea, which may release a wide range of air pollutants into the atmosphere. These air pollutants may affect the respiratory health of communities living close to the industrial areas. In particular, children are potentially more vulnerable than adults to environmental risk factors like air pollution. Unlike adults, the children’s respiratory system is constantly growing and they breathe more air than adults do, in proportion to their weight. During the stages of rapid growth, immature lungs may be the most susceptible [33,39,40]. Several studies have demonstrated the association between respiratory health such as asthma and lung function in children and proximity to petrochemical sites [41,42,43]. According to the study of Rusconi et al. children living in a petrochemical polluted area (Sarroch) versus a reference area (Burcei) showed a decrease in lung function (variation in FEV_1_ = −10.3% (90% CI = −15.0 to −6.0%)) [43].

To improve the accuracy and better reflect the linkages between air pollution and health outcomes, multiple methods for estimating exposure to ambient ozone were used. Use of spatial interpolation methods may be useful in providing individual-level exposures for locations without monitors and alternative for epidemiological analyses at the individual-level on the health effects. However, these approaches can contribute to uncertainty in estimating exposure, including monitoring site location, distance between a monitor and population, time-activity patterns of residents, and methodological choices. Because people spend more time indoors than outdoors, exposure misclassification bias can occur. For example, the average time spent per day in residential indoors was over 50% in 24 hours a day [44,45,46]. Thus, information on time spent in each micro-environment should be used in assessing actual personal exposures.

## 5. Conclusions

This study presents the association between ozone and lung function using spatial interpolation methods to estimate ozone exposure, and investigate how different methods for estimating exposure may influence health outcome for a cohort living near an industrial complex (Gwangyang Bay) in South Korea in 2009. We found an association between ozone and lung function, and a significant negative association was only observed in children by each spatial interpolation method. Our study illustrates that the kriging method provided more accurate estimates of exposure, and the effect size on lung function was generally higher when using the kriging approach. These results can be explained by the fact that the exposure estimation method used in estimating ozone exposure may affect the health outcome of epidemiologic studies. Future studies will be required to consider methodological choice with monitoring site locations, and time-activity patterns with exposure modeling estimates.

## Figures and Tables

**Table 1:** Summary statistics comparing the result of the four different methods for daily O_3_ concentrations at the the Gwangyang Bay industrial complex (*n* = 2283).

Model	Mean	SD	Min	25th	50th	75th	Max
Method 1	42.2	15.5	10.4	30.1	39.4	54.0	88.6
Method 2	40.9	16.6	3.7	28.9	38.9	51.8	113.8
Method 3	41.4	15.6	3.8	29.7	39.0	52.2	113.1
Method 4	41.7	15.9	5.9	29.6	39.2	52.6	113.5

Daily 8 h maximum moving average, Method 1: simple averaging, Method 2: nearest neighbor, Method 3: inverse distance weighting, Method 4: kriging.

**Table 2:** Distributions of O_3_ concentrations measured on lung function test day, 1 day, 2 days, 0–1 day, 1–2 days and 0–2 days by the four different methods.

Lag Days	Method 1		Method 2		Method 3		Method 4	
	Mean (SD)	Range	Mean (SD)	Range	Mean (SD)	Range	Mean (SD)	Range
0 day	59.8 (12.0)	31.5–85.9	56.0 (14.6)	26.0–85.7	57.8 (12.6)	30.2–93.9	58.1 (13.0)	31.5–97.8
1 day	58.1 (11.7)	35.8–82.8	53.9 (13.6)	26.0–93.9	55.4 (12.1)	30.9–93.4	55.7 (12.2)	30.0–98.2
2 days	58.9 (12.2)	28.3–85.0	56.2 (12.3)	24.0–96.4	57.7 (12.5)	24.0–90.5	57.9 (12.3)	23.9–90.1
0–1 day	58.9 (10.6)	36.7–84.4	54.9 (12.7)	30.0–95.4	56.6 (10.8)	33.5–93.6	56.9 (11.0)	36.3–91.6
1–2 days	58.5 (10.7)	32.0–81.8	55.0 (11.2)	26.8–93.6	56.6 (10.9)	27.9–89.6	56.8 (10.8)	27.3–88.3
0–2 days	58.9 (9.8)	36.2–83.2	55.3 (10.7)	34.1–90.7	57.0 (9.8)	34.7–89.0	57.2 (9.9)	34.7–87.2

Method 1: simple averaging, Method 2: nearest neighbor, Method 3: inverse distance weighting, Method 4: kriging, SD: standard deviation, 0 day: lung function test day, 1 day: 1 day before lung function test, 2 days: 2 days before lung function test, 0–1 day: average of the test day and previous day, 1–2 days: average of the last 2 days, 0–2 days: average of the test day and last 2 days.

**Table 3:** Results from cross-validation data in this study.

Pollutant	Method	RMSE	COD
O_3_	Nearest monitor	10.5	0.139
O_3_	IDW	8.75	0.113
O_3_	Kriging	7.48	0.099

RMSE: root mean square error, COD: coefficient of divergence.

**Table 4:** Demographic characteristics of the study subjects.

Variables		All	Age 9–14	Age 15–64	Age ≥ 65
*n*		2283	200	1419	664
Age (years)		41.8 ± 25.7	12.8 ± 1.23	30.7 ± 17.8	74.2 ± 5.60
Gender (%)	Male	43.1	47.5	46.5	34.5
	Female	56.9	52.5	53.5	65.5
Height (cm)		158.7 ± 10.0	145.4 ± 8.50	162.3 ± 8.57	155.2 ± 8.52
Weight (kg)		55.6 ± 12.3	37.8 ± 9.89	57.4 ± 11.6	57.1 ± 9.90
BMI (kg/m^2^)		21.9 ± 3.89	17.6 ± 3.53	21.7 ± 3.70	23.6 ± 3.25
Lung function	FEV1 (L)	2.37 ± 0.73	1.92 ± 0.35	2.69 ± 0.66	1.82 ± 0.55
	FVC (L)	2.90 ± 0.84	2.26 ± 0.42	3.23 ± 0.76	2.39 ± 0.71
	FEV1/FVC (%)	81.6 ± 8.76	85.5 ± 6.57	83.4 ± 7.65	76.6 ± 9.48

BMI: body mass index, FVC: forced vital capacity, FEV_1_: forced expiratory flow in 1 s.

**Table 5:** Age-specific effect of change in FVC (L) per increase IQR ^†^ of O_3_ concentration by various lags and four methods for estimating exposure.

Lag Day	Age	FVC (L)							
		Method 1		Method 2		Method 3		Method 4	
		β	(95% CI)	β	(95% CI)	β	(95% CI)	β	(95% CI)
0 day	9–14	−0.12	(−0.21, −0.03) *	−0.00	(−0.07, 0.07)	−0.01	(−0.07, 0.06)	−0.02	(−0.10, 0.06)
	15–64	−0.02	(−0.06, 0.03)	0.01	(−0.03, 0.05)	0.01	(−0.02, 0.05)	0.02	(−0.02, 0.07)
	≥65	−0.04	(−0.10, 0.01)	−0.03	(−0.09, 0.03)	−0.02	(−0.07, 0.03)	−0.03	(−0.09, 0.04)
	All	0.03	(−0.01, 0.07)	−0.00	(−0.04, 0.03)	0.02	(−0.01, 0.05)	0.03	(−0.01, 0.06)
1 day	9–14	−0.09	(−0.15, −0.03) *	−0.08	(−0.13, −0.03) *	−0.09	(−0.14, −0.04) *	−0.11	(−0.17, −0.04) *
	15–64	−0.04	(−0.09, 0.01)	0.01	(−0.03, 0.05)	−0.02	(−0.06, 0.03)	−0.02	(−0.08, 0.03)
	≥65	0.00	(−0.05, 0.06)	−0.03	(−0.08, 0.03)	−0.00	(−0.05, 0.04)	−0.01	(−0.07, 0.05)
	All	0.04	(−0.00, 0.07)	0.03	(−0.00, 0.07)	0.03	(−0.01, 0.06)	0.04	(0.00, 0.09)
2 days	9–14	−0.10	(−0.15, −0.05) **	−0.11	(−0.16, −0.06) **	−0.09	(−0.13, −0.05) **	−0.11	(−0.16, −0.06) **
	15–64	0.02	(−0.03, 0.07)	−0.01	(−0.06, 0.04)	−0.01	(−0.05, 0.03)	0.02	(−0.04, 0.08)
	≥65	−0.01	(−0.06, 0.05)	−0.03	(−0.10, 0.04)	−0.01	(−0.06, 0.04)	−0.01	(−0.07, 0.05)
	All	0.07	(0.03, 0.10)	0.05	(0.01, 0.09)	0.04	(0.01, 0.07)	0.07	(0.03, 0.12)
0–1 day	9–14	−0.11	(−0.19, −0.04) *	−0.06	(−0.13, 0.00)	−0.07	(−0.14, −0.01) *	−0.09	(−0.17, −0.01) *
	15–64	−0.04	(−0.09, 0.02)	0.01	(−0.03, 0.06)	0.00	(−0.04, 0.05)	0.01	(−0.05, 0.06)
	≥65	−0.02	(−0.08, 0.03)	−0.03	(−0.10, 0.03)	−0.01	(−0.06, 0.04)	−0.02	(−0.09, 0.05)
	All	0.04	(−0.00, 0.08)	0.02	(−0.02, 0.06)	0.03	(−0.01, 0.07)	0.05	(−0.00, 0.09)
1–2 days	9–14	−0.11	(−0.17, −0.06) **	−0.11	(−0.16, −0.06) **	−0.10	(−0.15, −0.05) **	−0.12	(−0.18, −0.07) **
	15–64	−0.02	(−0.07, 0.04)	0.00	(−0.05, 0.06)	−0.02	(−0.07, 0.03)	−0.00	(−0.07, 0.06)
	≥65	−0.00	(−0.060, 0.057)	−0.04	(−0.11, 0.03)	−0.01	(−0.06, 0.04)	−0.01	(−0.08, 0.06)
	All	0.07	(0.02, 0.11)	0.05	(0.01, 0.10)	0.05	(0.01, 0.08)	0.07	(0.02, 0.12)
0–2 days	9–14	−0.13	(−0.20, −0.06) **	−0.11	(−0.18, −0.04) *	−0.12	(−0.18, −0.05) **	−0.14	(−0.22, −0.07) **
	15–64	−0.03	(−0.09, 0.04)	0.01	(−0.05, 0.06)	−0.00	(−0.06, 0.05)	0.02	(−0.05, 0.08)
	≥65	−0.02	(−0.08, 0.04)	−0.04	(−0.12, 0.03)	−0.01	(−0.07, 0.04)	−0.02	(−0.09, 0.05)
	All	0.07	(0.02, 0.11)	0.04	(−0.01, 0.08)	0.05	(0.01, 0.09)	0.07	(0.02, 0.12)

^†^: IQR is 17.3 ppb for O_3_, FVC: forced vital capacity, Method 1: simple averaging, Method 2: nearest neighbor, Method 3: inverse distance weighting, Method 4: kriging, CI: confidence interval, 0 day: lung function test day, 1 day: 1 day before lung function test, 2 days: 2 days before lung function test, 0–1 day: average of the test day and previous day, 1–2 days: average of the last 2 days, 0–2 days: average of the test day and last 2 days. **: *p* < 0.001, *: *p* < 0.05.

**Table 6:** Age-specific effect of change in FEV_1_ (L) per increase IQR ^†^ of O_3_ concentration by various lags and four methods for estimating exposure.

Lag Day	Age	FEV_1_ (L)							
		Method 1		Method 2		Method 3		Method 4	
		β	(95% CI)	β	(95% CI)	β	(95% CI)	β	95% CI
0 day	9–14	−0.12	(−0.20, −0.04) *	−0.02	(−0.08, 0.04)	−0.03	(−0.08, 0.03)	−0.04	(−0.11, 0.03)
	15–64	−0.00	(−0.04, 0.04)	0.02	(−0.01, 0.05)	0.03	(−0.00, 0.06)	0.04	(0.00, 0.08)
	≥65	−0.07	(−0.12, −0.03) *	−0.07	(−0.11, −0.02) *	−0.06	(−0.10, −0.02) *	−0.07	(−0.12, −0.02) *
	All	0.02	(−0.01, 0.06)	−0.01	(−0.04, 0.02)	0.02	(−0.01, 0.04)	0.02	(−0.01, 0.05)
1 day	9–14	−0.09	(−0.14, −0.04) *	−0.07	(−0.12, −0.03) *	−0.08	(−0.12, −0.04) *	−0.10	(−0.15, −0.04) *
	15–64	−0.03	(−0.08, 0.01)	0.02	(−0.02, 0.06)	−0.00	(−0.04, 0.03)	−0.01	(−0.06, 0.04)
	≥65	−0.02	(−0.07, 0.02)	−0.02	(−0.07, 0.03)	−0.02	(−0.06, 0.02)	−0.02	(−0.07, 0.02)
	All	0.02	(−0.01, 0.06)	0.04	(0.00, 0.07)	0.03	(−0.00, 0.05)	0.04	(0.00, 0.07)
2 days	9–14	−0.08	(−0.12, −0.04) *	−0.08	(−0.13, −0.04) *	−0.07	(−0.11, −0.03) *	−0.08	(−0.13, −0.04) *
	15–64	0.05	(0.00, 0.09)	0.02	(−0.02, 0.07)	0.02	(−0.02, 0.06)	0.05	(0.00, 0.10)
	≥65	−0.05	(−0.09, −0.01) *	−0.05	(−0.11, 0.00)	−0.04	(−0.08, −0.00) *	−0.05	(−0.10, −0.00) *
	All	0.06	(0.03, 0.09)	0.06	(0.02, 0.09)	0.04	(0.01, 0.07)	0.06	(0.03, 0.10)
0–1 day	9–14	−0.11	(−0.18, −0.04) *	−0.06	(−0.12, −0.01) *	−0.08	(−0.13, −0.02) *	−0.10	(−0.16, −0.03) *
	15–64	−0.02	(−0.07, 0.03)	0.02	(−0.02, 0.06)	0.02	(−0.02, 0.06)	0.03	(−0.02, 0.08)
	≥65	−0.06	(−0.10, −0.01) *	−0.06	(−0.11, −0.00) *	−0.04	(−0.09, 0.00)	−0.06	(−0.11, −0.00) *
	All	0.03	(−0.01, 0.07)	0.02	(−0.02, 0.05)	0.03	(−0.01, 0.06)	0.04	(−0.00, 0.08)
1–2 days	9–14	−0.10	(−0.15, −0.05) **	−0.09	(−0.14, −0.04) *	−0.08	(−0.13, −0.04) **	−0.10	(−0.15, −0.05) **
	15–64	0.01	(−0.04, 0.06)	0.03	(−0.02, 0.08)	0.01	(−0.03, 0.05)	0.03	(−0.03, 0.08)
	≥65	−0.04	(−0.09, 0.00)	−0.05	(−0.11, 0.01)	−0.04	(−0.08, 0.01)	−0.05	(−0.10, 0.01)
	All	0.05	(0.02, 0.09)	0.06	(0.02, 0.10)	0.04	(0.01, 0.07)	0.06	(0.02, 0.10)
0–2 days	9–14	−0.12	(−0.18, −0.06) **	−0.10	(−0.16, −0.04) *	−0.10	(−0.16, −0.05) *	−0.12	(−0.19, −0.06) **
	15–64	0.01	(−0.05, 0.06)	0.03	(−0.01, 0.08)	0.03	(−0.02, 0.08)	0.05	(−0.01, 0.11)
	≥65	−0.06	(−0.11, −0.01) *	−0.07	(−0.13, −0.01) *	−0.05	(−0.09, −0.00) *	−0.06	(−0.12, −0.01) *
	All	0.05	(0.01, 0.09)	0.04	(−0.00, 0.08)	0.04	(0.01, 0.08)	0.06	(0.02, 0.11)

^†^: IQR is 17.3 ppb for O_3_, FEV_1_: forced expiratory flow in 1 s, Method 1: simple averaging, Method 2: nearest neighbor, Method 3: inverse distance weighting, Method 4: kriging, 0 day: lung function test day, 1 day: 1 day before lung function test, 2 days: 2 days before lung function test, 0−1 day: average of the test day and previous day, 1−2 days: average of the last 2 days, 0−2 days: average of the test day and last 2 days. **: *p* < 0.001, *: *p* < 0.05.
